# Exploring the Complexities of Non-Bacterial Thrombotic Endocarditis: Highlights from Literature and Case Studies

**DOI:** 10.3390/jcm13164904

**Published:** 2024-08-20

**Authors:** Giuseppe Santarpino, Francesca Lofrumento, Concetta Zito, Olimpia Trio, Davide Restelli, Maurizio Cusmà Piccione, Roberta Manganaro, Scipione Carerj, Francesco Cardetta, Corrado Fiore, Cesare de Gregorio

**Affiliations:** 1 Department of Cardiac Surgery, Magna Graecia University, 88100 Catanzaro, Italy; 2 Department of Cardiac Surgery, Città di Lecce Hospital, GVM Care and Research, 73100 Lecce, Italy; cardiologo85@gmail.com; 3 Department of Cardiac Surgery, Paracelsus Medical University, 40100 Nuremberg, Germany; 4 Department of Clinical and Experimental Medicine, G. Martino University Hospital, Cardiology Unit, 98122 Messina, Italy; franci.lofru@gmail.com (F.L.); concetta.zito@unime.it (C.Z.); olimpia.trio@gmail.com (O.T.); dv.restelli@gmail.com (D.R.); maurizio.cusma@gmail.com (M.C.P.); manganaro.roberta@gmail.com (R.M.); scipione.carerj@unime.it (S.C.); cesare.degregorio@unime.it (C.d.G.); 5 Department of Cardiac Surgery, Campus Biomedico University, 00128 Rome, Italy; f.cardetta@unicampus.it; 6 Department of Emergency, G. Martino University Hospital, Cardiology Unit, 98122 Messina, Italy

**Keywords:** non-bacterial thrombotic endocarditis, multimodality imaging, differential diagnosis, tailored therapy, valvular heart disease

## Abstract

Non-bacterial thrombotic endocarditis (NBTE) is a form of non-infective endocarditis characterized by the deposition of sterile fibrin and platelets on cardiac valves. Even though some studies have identified important pathophysiological features, many aspects remain poorly understood. Given its wide availability, transthoracic echocardiography is typically the initial diagnostic approach to the patient. Additionally, recent technological advancements in transesophageal echocardiography, such as three-dimensional and multiplanar reconstruction analysis, have significantly improved diagnostic accuracy over time. By presenting our case series and performing a literature review, we focused on the main pathophysiologic, diagnostic, and therapeutic aspects of this rare but potentially life-threatening disease.

## 1. Introduction

First identified by Ziegler in 1888, non-bacterial thrombotic endocarditis (NBTE), a term coined in 1936 by Gross and Friedberg [1], known as marantic, verrucous, or Libman–Sacks endocarditis [2], is a form of sterile endocarditis with deposition of fibrin and platelets on cardiac valves [3,4,5,6].

For many years, it was an underdiagnosed condition mostly found at autopsy [6,7]. Currently, a premortem diagnosis has become feasible thanks to the rapid evolution of cardiovascular imaging techniques. Multimodality imaging, notably including echocardiography and positron emission tomography/computed tomography (PET/CT), holds significant potential for earlier diagnosis, better risk stratification, and clinical management than in the past [4,5,6].

By analyzing a personal case series and current literature, this review aims to shed light on this rare but potentially life-threatening condition.

## 2. Epidemiology and Etiology

Most of available data concerning NBTE stem from case reports and post-mortem investigations [6,7,8]. NBTE is a rare condition, with a reported incidence ranging between 1.1% and 1.6% in autopsy studies [8,9,10]. Bussani et al. recently conducted the most extensive post-mortem cohort studies to date, revealing an increased prevalence of NBTE at approximately 3.7% [7]. In fact, within this cohort, while the diagnosis of infective endocarditis (IE) predominantly relied on clinical evaluations, none of the 405 NBTE cases had been identified prior to death. This observation underscores the ambiguity surrounding the clinical incidence and prevalence of NBTE, contrasting with the recognized incidence of IE [8].

Both clinical investigations and post-mortem analyses have extensively evidenced the correlation between NBTE and specific underlying conditions, notably cancer, autoimmune disorders, and hypercoagulable states [3,4,5,6,7]. In a recent contemporary large cohort study, it was observed that 41% of patients diagnosed with NBTE had concomitant cancer, 36% exhibited antiphospholipid antibodies syndrome (APLs), and 33% had systemic lupus erythematosus (SLE), with 21% presenting both SLE and APLs concurrently [6]. The predominant malignancies identified were lung adenocarcinomas (47.1%), breast malignancies (23.5%), and pancreatic malignancies (17.6%) [6]. Moreover, an association is also reported between NBTE and hypercoagulable conditions, such as disseminated intravascular coagulation, primary antiphospholipid syndrome (APLs), burns, and sepsis [3,4,5,6,7]. In rare instances, NBTE may arise as a complication of systemic infections, such as tuberculosis and human immunodeficiency virus. Finally, sporadic cases occurred in patients with rheumatoid arthritis, and associations between NBTE and less common autoimmune conditions, such as granulomatosis with polyangiitis, Behçet’s disease, and adult-onset Still’s disease, have been highlighted in isolated case reports [3,5,10].

The highest prevalence is observed among individuals aged 40 to 80 years, while susceptibility spans across all age demographics, without any apparent gender bias [3,4]. However, a recent investigation showed a higher prevalence in women probably linked to the elevated incidence of connective tissue diseases within the study cohort, which are recognized to occur more commonly in women than in men and likely contribute to this observed gender discrepancy [11].

## 3. Pathophysiology

Sterile fibrin–platelet thrombi on cardiac valves are the pathological hallmark of NBTE, resulting from endothelial damage and exposure of the subendothelial connective tissue to circulating platelets. NBTE pathogenesis remains inadequately elucidated, but there are several interacting mechanisms, including circulating immune complexes, hypercoagulable states, and carcinomatosis, potentially contributing to its manifestation [3,4,5,6,11].

### 3.1. Immune Complexes

A potential pathogenic role of circulating immune complexes in NBTE has been suggested since 1980 by Williams et al. [12]. Subsequently, immunoglobulin and complement complexes deposition have been demonstrated along the edges of valve leaflets and within valvular vegetations in patients diagnosed with SLE using immuno-histochemical techniques. So, immune complexes appear to play a pivotal role in mediating valvular tissue injury both in patients with SLE and secondary APLs [3,5,13,14].

However, NBTE may also manifest in cases of primary APLs [15]. In fact, initially believed to induce a hypercoagulable state leading to thrombus formation, it is now acknowledged that primary APLs may also contribute to valve damage through direct immune-mediated reactions, especially through autoantibodies, i.e., anticardiolipin antibodies, directed against negatively charged phospholipids in endothelial cell membranes [5,16].

### 3.2. Hypercoagulable States

The hypothesis that valvular degeneration and hypercoagulability play a crucial role in the development of NBTE dates back as far as 1956 by MacDonald and Robbins [17]. Currently, the association between NBTE and hypercoagulable conditions, such as primary APLs, malignancy, and DIC, is well known [3,5,18,19,20,21,22]. Although the precise triggering factors remain unidentified, it is plausible that endothelial injury serves as the underlying basis for the deposition of platelets and fibrin within an already predisposed prothrombotic environment [5].

### 3.3. Carcinomatosis

The correlation between carcinomatosis and thrombosis, first described in 1865 by Trousseau [23], has been firmly established in the literature [3,5,10,16,24]. This phenomenon arises from a combination of various overlapping and interacting mechanisms. First, the interaction between macrophages and malignant cells stimulates the production of circulating cytokines, including tumor necrosis factor and interleukin 1, which induce endothelial damage, subsequently promoting sterile thrombi formation and platelet aggregation [5,24]. Moreover, the expression of tissue factor on tumor cell surfaces enhances thrombin production through extrinsic coagulation pathway activation. A cysteine protease, known as “cancer procoagulant”, has also been documented to directly activate factor X independently of factor VII. Synthesis of procoagulant factors, like tissue factor and plasminogen activator inhibitor 1, may also be induced by hypoxic conditions within the tumor microenvironment and by upregulation of the MET oncogene. In addition, the association between mucin-producing adenocarcinoma and NBTE is widely recognized; mucins can interact with P- and L-selectins on platelet and endothelial surface, triggering platelet-rich microthrombi formation [24]. Additionally, anaphylaxis and toxicity to various substances, including chemotherapy agents, may induce substantial cytokine release and immune complex production causing a wide tissue damage, including heart valve structures, through a direct or indirect inflammatory mechanism [25]. Finally, coagulation abnormalities consistent with disseminated intravascular coagulation may particularly manifest in more advanced malignancy, further exacerbating the hypercoagulable state [5].

## 4. Clinical Presentation

Clinical suspicion is fundamental for an early diagnosis of NBTE, particularly through a rapid identification of the underlying predisposing conditions (Graphical abstract). In fact, patients typically remain asymptomatic until advanced complications arise, such as embolization or valvular dysfunction. In a recent contemporary Cleveland Clinic registry [6], stroke emerged as the most prevalent clinical presentation upon admission (60%), followed by heart failure (21%) and acute coronary syndrome (7%), consistent with existing literature data [3,5,8,26,27]. NBTE vegetations exhibit greater friability and susceptibility to embolism in comparison with those observed in infective endocarditis, due to the absence of inflammation at the valve attachment site [28]. Embolization to the central nervous system, kidneys, extremities, spleen, and coronary arteries are the most commonly reported lesions [3,5,14,29].

## 5. Personal Case Contribution

We present three personal cases exemplifying the main clinical presentations of NBTE: embolic stroke, occasional finding in patient with predisposing conditions, and heart failure, respectively.

### 5.1. Case 1

**Figure 1:**
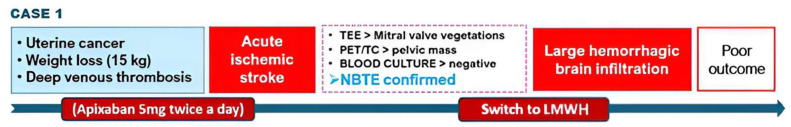
Timeline summarizing the main clinical events from Case 1, chronologically described.

A 56-year-old female presented to the emergency department with left hemiplegia and buccal rim deviation. Her past medical history included uterine cancer treated with hysterectomy, twofold occurrence of deep vein thrombosis on Apixaban therapy, and a significant weight loss of about 15 kg in few months (Figure 1).

**Figure 2:**
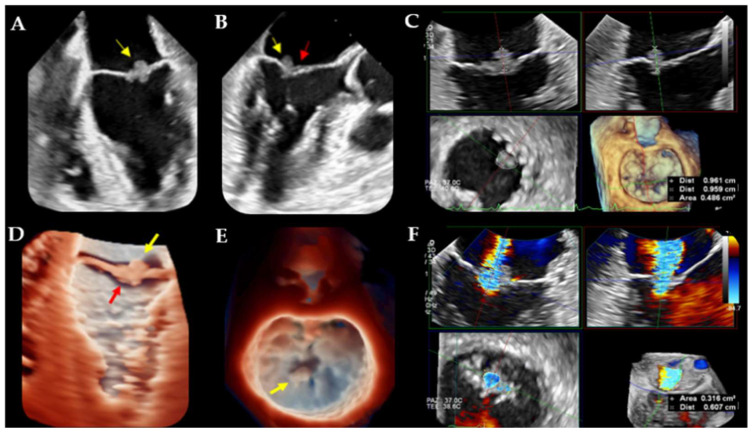
(**A**,**B**) Mid-esophageal transesophageal echocardiogram (TEE) views showing an iso-echogenic mass on P2 scallop tip (yellow narrow) and a thickened A2 edge (red narrow). (**C**) Three-dimensional TEE multiplanar reconstruction focused on P2 lesion size: 9.6 mm in length, area of 48 mm^2^. (**D**,**E**) Three-dimensional TEE-true view technique—a live 3D TEE (**E**) and a 3D “zoom” surgical view—(**F**) showing mitral valve lesions on both atrial (yellow narrows) and ventricular sides (red narrow). (**F**) A moderate mitral valve regurgitation in a 3D TEE multiplanar reconstruction: Proximal Isovelocity Surface Area (PISA) radius = 6 mm, VCA (vena contracta area) 3D = 0.3 cm^2^.

On admission, a brain CT scan and magnetic resonance imaging (MRI) revealed an acute ischemic stroke of right middle cerebral artery immediately treated with mechanical thrombectomy. Subsequently, transthoracic echocardiography (TTE) was conducted to investigate potential cardioembolic sources, revealing a small iso-echogenic mass at the tip of the posterior mitral valve leaflet associated with moderate regurgitation. An in-depth transesophageal echocardiogram (TEE) examination showed two lesions: a soft, iso-echogenic, highly mobile mass at the tip of the P2 scallop; and a thickened anterior edge, which was assumed to be the anchor site for a second mass to subsequently embolize (Figure 2).

**Figure 3:**
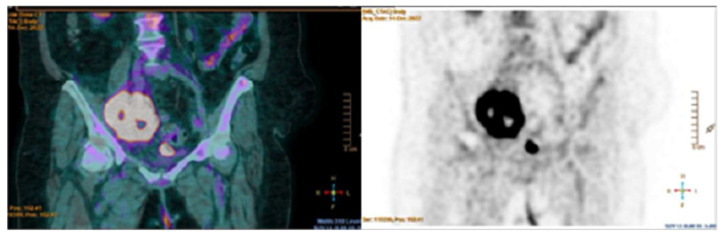
A large mass in the right pelvic region exhibiting widespread and non-uniform ^18^F-FDG on the PET/CT scan.

The patient was afebrile and the clinical examination revealed no significant findings. Serial blood cultures, serology for intra-cellular organisms, and testing for autoimmune disease and coagulopathy were negative, so a diagnosis of marantic endocarditis was made. A PET/CT scan was then performed unveiling a large ^18^F-fluorodeoxyglucose (FDG)-uptaken mass in the right pelvic region, in contrast to the cardiac area (Figure 3). An immediate switch from Apixaban to low-molecular-weight heparin (LMWH) was deemed necessary, also due to an ongoing hemorrhagic trend in the recent ischemic stroke area, precluding any consideration for cardiac surgery.

### 5.2. Case 2

**Figure 4:**
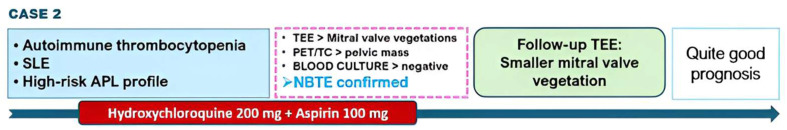
Event timeline from case 2.

A 60-year-old female patient affected by SLE and high-risk APLs was referred to our cardiology unit for routine cardiological work up and an autoimmune thrombocytopenia, requiring the initiation of hydroxychloroquine therapy [30] (Figure 4).

**Figure 5:**
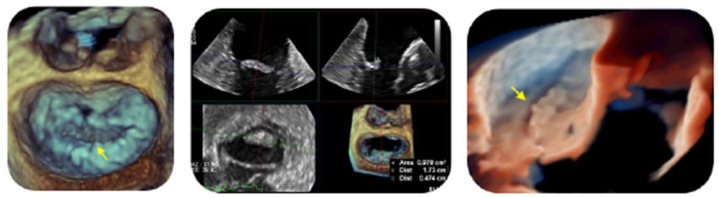
On the left, the huge echo-dense mass along the free edge of the P2 scallop (yellow narrow) in a 3D “zoom” surgical view and in a 3D TEE multiplanar reconstruction, in the middle, measuring 17 × 4.7 mm and covering an area of 98 mm^2^. On the right, NBTE vegetation (yellow narrow) seen in a 3D “zoom” TEE view (lateral perspective-true view technique).

**Figure 6:**
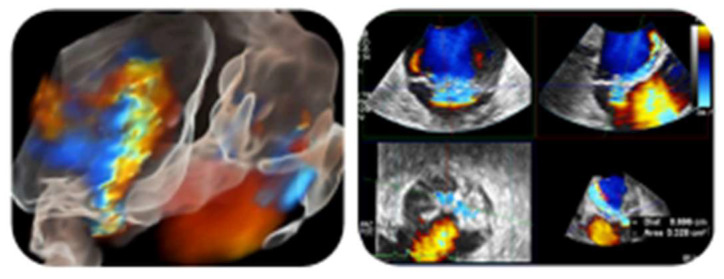
On the right, a 3D TEE multiplanar reconstruction showing a moderate mitral valve regurgitation (PISA radius = 6.9 mm, VCA 3D = 0.328 cm^2^] with an eccentric anterior direction, as seen on the left in a lateral perspective 3D “zoom” TEE acquisition—glass technique.

TTE revealed an eccentric mitral valve regurgitation, further investigated with TEE, that showed a huge echo-dense mass along the P2 scallop tip (Figure 5), causing a moderate mitral regurgitation (Figure 6).

Considering the absence of infectious events and clinical manifestations (no fever, no suspicious skin lesions), along with negative serial blood cultures and systematic serological testing, the diagnosis of Libman–Sacks endocarditis was established. Due to the patient’s high-risk APLs profile and thrombocytopenia (platelet count 50.000/μL), therapy with aspirin at 100 mg daily was preferred, based on current guidelines [30,31].

**Figure 7:**
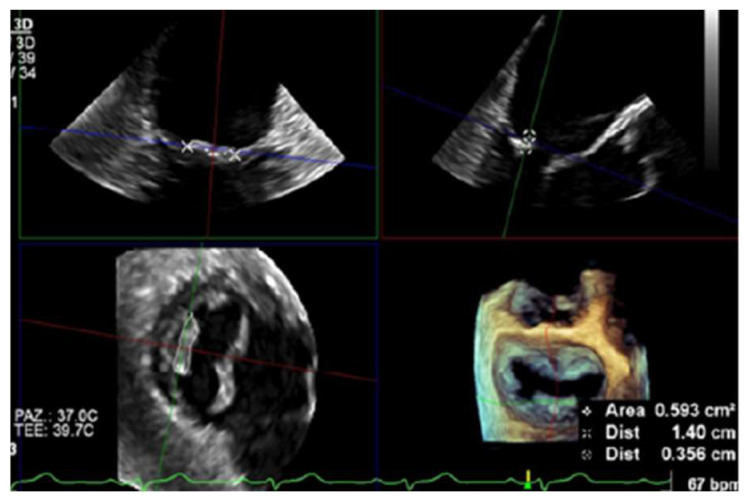
Twelve-month TEE follow-up showing a significant decrease in NBTE lesion: 14 × 3.5 mm, area of 0.59 cm^2^ in a 3D TEE multiplanar reconstruction.

The patient luckily experienced uneventful follow up. A six- and twelve-month TEE revealed a notable decrease in the size of the mass (Figure 7), with a stable moderate mitral regurgitation. 

### 5.3. Case 3

**Figure 8:**
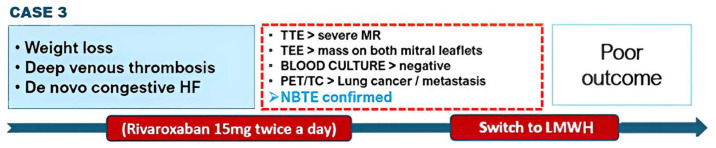
Event timeline from Case 3.

An 82-year-old male patient was admitted to our cardiology department with newly diagnosed acute congestive heart failure. Past medical history comprised a weight loss of approximately 10 kg over the last 2 months and two episodes of deep vein thrombosis on rivaroxaban therapy (Figure 8).

**Figure 9:**
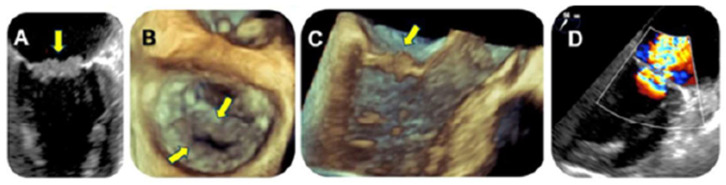
(**A**) A 60° mid-esophageal TEE view showing a verrucous big mass on the mitral valve (yellow narrow). (**B**,**C**) Verrucous vegetations (yellow narrows) along the entire coaptation surface of both mitral leaflets in 3D “zoom” TEE acquisitions. (**D**) The severe mitral regurgitation on 2D TEE.

A TTE revealed thickened mitral valve leaflets with a severe mitral regurgitation. A subsequent TEE showed a verrucous, hypoechogenic mass along the entire mitral leaflets coaptation surface (Figure 9). 

**Figure 10:**
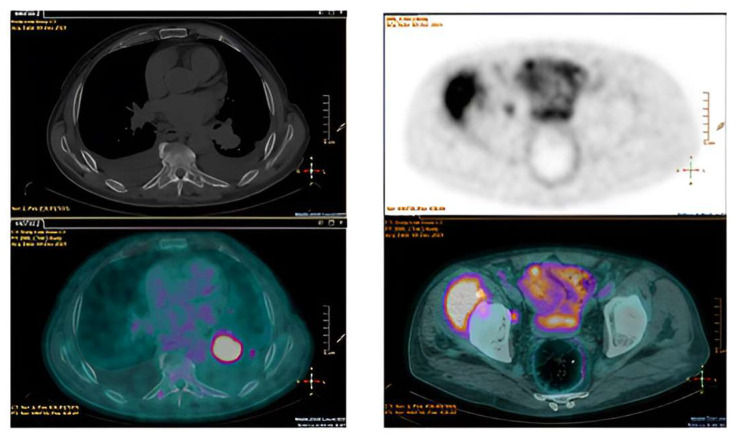
^18^F–FDG–PET/CT scan revealing a significantly abnormal metabolic uptake within a left pulmonary lesion, on the left, also involving adrenal glands, lymph nodes and bone sites, on the right.

No fever or other signs of infection were observed. Work-up for autoimmune diseases or coagulopathy was negative, as well as all serial blood cultures and serology screening. So, LMWH was initiated, replacing rivaroxaban. A ^18^F–FDG–PET/CT scan revealed abnormal metabolic uptake in a left pulmonary nodule, with elevated uptake observed at lymph nodes, adrenal glands, and bone sites too (Figure 10). Therefore, NBTE was confirmed as an epiphenomenon of the tumor disease in its metastatic phase, unfortunately with a poor prognosis.

### 5.4. Case Discussion

From the presentation of these cases, several key points can be identified:

- Previous deep venous thrombosis and weight loss could be considered as red flags for marantic NBTE (as in cases 1 and 3);
- The fundamental role of multimodality imaging (TTE + TEE, brain MRI, ^18^F-FDG/PET–CT scan) for NBTE diagnosis;
- The role of 2D and 3D-TEE for detection and characterization of NBTE valvular lesions;
- Failure of direct oral anticoagulants (DOACs) in NBTE setting (as in cases 1 and 3);
- The need for a tailored antithrombotic therapy;
- Efficacy of 100 mg aspirin in reducing NBTE lesions (case 2: Libman–Sacks endocarditis, high-risk APLs profile, and thrombocytopenia).

These aspects will be further examined in the subsequent sections, where we will discuss NBTE diagnosis and treatment based on the existing literature and the insights provided by the cases presented.

## 6. Diagnosis

**Figure 11:**
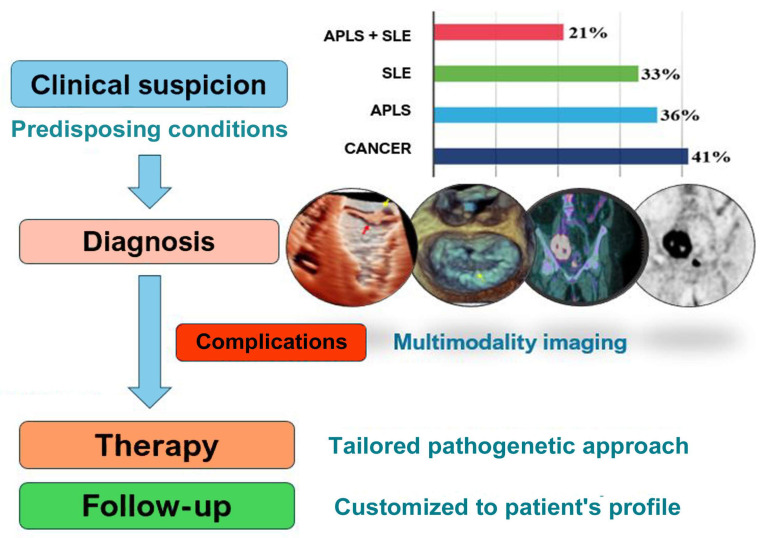
A simplified flow-chart for NBTE management work-up.

By integrating our experience with current evidence from the literature, a simplified flow-chart is presented to help clinicians in NBTE management (Figure 11).

### 6.1. Laboratory Investigations

Firstly, it is worth it to rule out IE (Table 1). So, it is recommended to obtain a minimum of three sets of blood cultures before starting empiric antibiotic therapy, together with serology and Polymerase Chain Reaction (PCR) of explanted valve to mitigate the occurrence of negative blood cultures, observed in up to 30% of IE cases as a result of prior antibiotic therapy or involvement of fastidious bacteria (e.g., fungi, HACEK [*Haemophilus*, *Aggregatibacter*, *Cardiobacterium*, *Eikenella*, *Kingella*] group) or intracellular microorganisms (e.g., *Coxiella burnetii*, *Mycoplasma* spp., *Legionella* spp., *Tropheryma whipplei*) [3,5,8,32,33]. Therefore, according to the latest guidelines on IE, it is recommended to perform systematic serological testing for *C. burnetii*, *Bartonella* spp., *Aspergillus* spp., *L. pneumophila*, *Brucella* spp., and *M. pneumoniae*, followed by specific PCR assays from valvular tissue for *Tropheryma whipplei*, *Bartonella* spp., and fungi (*Candida* spp., *Aspergillus* spp.) in order to effectively differentiate between blood culture-negative infective endocarditis (BCNE) and NBTE [8,32,33].

Once both IE and BCNE have been excluded, supplementary laboratory investigations are needed to search for NBTE-predisposing conditions like autoimmune disorders (particularly LES and APLs), cancer, and hypercoagulability, through immunological assays (i.e., antinuclear antibody, extractable nuclear antigens, anti-double stranded DNA, rheumatoid factor, anticardiolipin, and anti-β2-glycoprotein 1 antibodies), tumor markers, coagulation profile (prothrombin time, partial thromboplastin time, fibrinogen, D-dimers, and cross-linked fibrin degradation products) [3,5,8,25].

However, it is important to note that coagulative disorders may not be specific markers of NBTE [8].

### 6.2. Multimodality Imaging

Echocardiography plays a pivotal role as a first imaging approach to NBTE, essential in the differential diagnosis with an infective form (Table 1), Lambl excrescences, fibroelastoma, or other benign masses [8].

Of note, NBTE may be shown with different morphology on echocardiography: from a diffuse valvular thickening to valvular vegetations [3,4,5,6,34]. Moreover, vegetations can exhibit considerable variability in their shape, being sessile, tubular, or coalescent (like ‘*kissing lesion*’ in Libman–Sacks endocarditis or marantic NBTE [35,36]), as well as echogenicity and motility. The typical aspect is a verrucous, broad-based, and irregular vegetation, generally located at the tips of valvular leaflets (Figure 2A–E, Figure 5 and Figure 9B,C), which may extend to their mid and basal regions and potentially involving both valvular sides (as in our Figure 2D) [6,8,34,37]. Vegetations can range from small sub-centimeter masses to large excrescences (greater than 2 cm) [6].

Typically, NBTE predominantly affects the left-sided heart valves, particularly the mitral valve (62% compared to 24% for the aortic valve) [6], with occasional involvement of the right heart valves and implanted devices [3,4,8,34].

Usually, NBTE vegetations do not result in a significant valve dysfunction, and local complications are rare, which may help in the differential diagnosis with IE (Table 1) [8].

However, it is crucial to emphasize that echocardiography may yield false-negative results if friable vegetations have already embolized [5,6,7,8] or in cases of very small vegetations, particularly those measuring less than 5 mm [3,4,5,6]. So, there is a large agreement recommending TEE for all patients with nondiagnostic TTE findings and a high clinical suspicion for NTBE [3,4,5,6,8], having confirmed its higher sensitivity (97.1%) compared to TTE (45.2%) [6,8]. In addition, 3D-TEE improves anatomical details compared to 2D-TEE imaging; for instance, it helps in identifying vegetations on mitral commissural scallops or involving both sides of mitral leaflets (Figure 2D) or aortic cusps [34,37]. Furthermore, the role of TTE in assessing cardiac volumes and function should not be overlooked [3].

Moreover, a multimodality imaging approach is crucial in NBTE diagnostic work-up (Graphical abstract) [38,39], as also emphasized in the current guidelines for IE [8].

Thanks to its better spatial resolution, cardiac CT (CCT) is recommended as a supplementary imaging technique to echocardiography [40,41,42]. It has a Class I recommendation and Level B evidence for detection of valvular lesion in case of possible native valve endocarditis (NVE) and, also, to diagnose paravalvular lesion in NVE and prosthetic valve endocarditis (PVE), particularly when echocardiography (TTE and TEE) yields inconclusive results [8]. However, the lower temporal resolution of cardiac CT compared to echocardiography can result in false-negative results, particularly with small (<1 cm) and mobile vegetations, which are commonly seen in NBTE where CCT can detect coronary embolism instead [40,42,43]. Finally, CCT may serve as a suitable alternative to invasive coronary angiography in patients undergoing cardiac surgery, in case of IE as well as of NBTE [8,44]. Additionally, the ability to extend the examination to the entire body (whole-body CT) provides significant advantages, such as detection of systemic complications, like cerebral or peripheral embolization, both in IE and NBTE, and potential identification of tumor lesions (primary and/or metastatic cancer) associated with NBTE [8,40,43,44].

Also, brain MRI is essential for cerebral embolism detection (Table 1) [3,5]. Furthermore, given that stroke is the most common clinical presentation upon admission, brain MRI often serves as the initial examination that triggers diagnostic suspicion of NBTE, with echocardiography following later in the diagnostic process, as in our Case 1 and in cases from the literature [14,18,45,46,47].

Additionally, ^18^F–FDG/PET–CT scans can detect primary tumor and/or metastases [39,46], thereby supporting the diagnosis of NBTE in the absence of infective triggers, as observed in our Cases 1 and 3. Of clinical note, NBTE lesions typically do not show ^18^F–FDG uptake in the cardiac area, thus ruling out IE (Table 1) [5,39,48].

## 7. Treatment

There is still no specific treatment for NBTE. Current management strategies are guided by two main objectives: firstly, to mitigate the risk of recurrences by treating predisposing conditions specifically, and secondly, to reduce the risk of systemic embolisms through antithrombotic therapy [3,4,5,8,16]. Surgical intervention is typically rare and reserved for specific cases [8,49].

### 7.1. Specific Treatment for the Predisposing Condition

Treating the predisposing conditions is pivotal in preventing recurrent NBTE [6,8,21,22,50,51]. Certainly, this treatment should be overseen by the appropriate specialist, such as an oncologist or rheumatologist, depending on the underlying disease. Unfortunately, most cases of NBTE are frequently observed in patients with advanced metastatic neoplasia, for whom only palliative therapy is indicated [4,5] (Case 1, Case 3). On the other hand, NBTE associated with SLE can manifest at any stage of the disease and seems to be independent of disease activity [3,5].

### 7.2. Antithrombotic Therapy

Given the high risk of embolism associated with NBTE friable vegetation, lifelong antithrombotic therapy is recommended for all patients, whether for preventive or therapeutic purposes, with a careful assessment of the individual’s bleeding risk [3,4,5,6,8], including also the risk for heparin-induced thrombocytopenia [52]. Anticoagulation can include LMWH, unfractionated heparin or vitamin K antagonists [8]. There is still no evidence supporting the beneficial use of direct oral anticoagulants (DOACs) in NBTE settings. Two randomized open-label non-inferiority studies comparing rivaroxaban to warfarin in patients with APLs indicated a higher risk of thromboembolic events and major bleeding associated with rivaroxaban [53,54]. Moreover, the literature cases, as well as our Case 1 and Case 3, emphasize the need for caution when considering the use of DOACs in patients with APLs or cancer [15,21,29,46,55,56,57].

Moreover, vitamin K antagonists were reported to be less effective than heparin for preventing thromboembolic events in cancer-related NBTE [6,24,56,58,59]. In fact, in addition to its inhibiting effect on factor Xa and thrombin, heparin has exhibited supplementary beneficial properties in cancer thrombosis; these include increased release of tissue factor pathway inhibitor from the vascular endothelium at sites of active thrombosis, binding and neutralization of tumor-derived inflammatory cytokines, and binding of L- and P-selectins, thereby preventing platelet-rich microthrombi formation (Section 3.3) [24,57].

**Figure 12:**
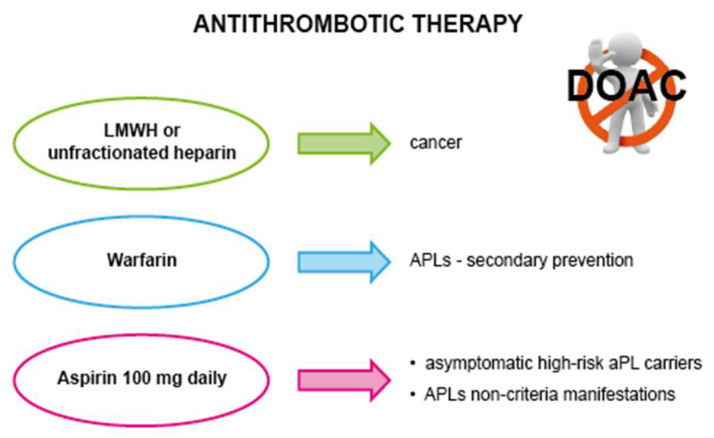
A tailored antithrombotic approach.

On the other hand, vitamin K antagonists represent the treatment of choice for patients with APLs who experience a thrombotic event [30,60,61]. Instead, a prophylactic regimen of aspirin 100 mg daily is recommended for asymptomatic aPL carriers with a high-risk profile [30,60]. A similar therapeutic approach is suggested for patients presenting with non-criteria manifestations of APLs, such as cardiac valve disease and thrombocytopenia, which may also coexist as in our Case 2 [31,62]. Therefore, low-dose aspirin may be considered as a preferable option for patients with mild to moderate thrombocytopenia and NBTE vegetations with a high thromboembolic risk [31] (Figure 12), also with a potential positive effect in mitigating the progression of NBTE vegetations, as illustrated in Case 2.

### 7.3. Surgery

The role of surgery for NBTE management is still controversial and requires further clarification [8].

As recommended for IE, surgery should be considered in NBTE patients with cardiogenic shock, established or recurrent thromboembolism, despite therapeutic anticoagulation, and large vegetations (≥10 mm) with a high embolic risk [5,8,16,44,49,63,64]. However, unlikely IE, valve-sparing surgery is often feasible (Table 1) [3,5,65].

A careful assessment of the individual risk–benefit balance is mandatory before considering a surgical approach, particularly in patients with life-threatening malignancy, as in our Case 1 and 3 [3,5,50].

## 8. Prognosis and Follow-Up

The prognosis is primarily dependent on the underlying condition, with significantly poorer outcomes in cases associated with metastatic disease (Cases 1 and 3) [3].

Currently, there are no guidelines for the follow-up of NBTE patients. Nevertheless, follow-up protocol should incorporate both clinical and imaging assessments [3,5]. It is crucial to rule out recurrent systemic embolism and ensure appropriate antithrombotic therapy while minimizing the risk of bleeding and thrombocytopenia as much as possible [3,5,66].

Regular TTE follow-up should be scheduled, based on the individual patient’s profile, to monitor NBTE lesions and valvular function over time [3,5].

## 9. A Specialized “NBTE Team” Could Be Useful?

**Figure 13:**
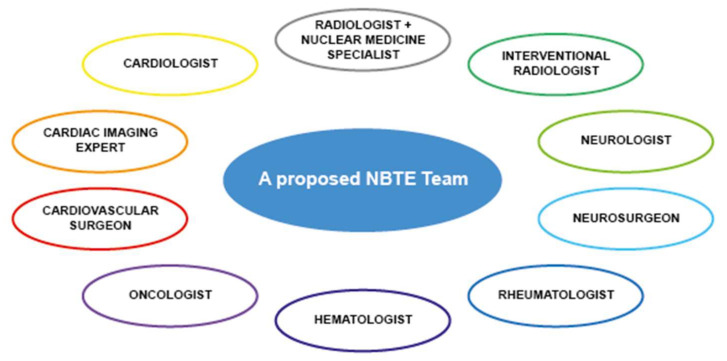
A proposed NBTE Team.

Although less common than IE, the management of NBTE can be even more intricate, as evidenced so far. Therefore, according to the most recent guidelines on IE [8], the establishment of a specialized “NBTE Team” should prove beneficial in managing this persistently challenging condition. The “NBTE team” should encompass a multidisciplinary group (Figure 13), including cardiologists, particularly those specializing in multimodality imaging, as well as cardiovascular surgeons, radiologists, and nuclear medicine experts. The inclusion of rheumatologists, hematologists, and oncologists could be essential in the management of predisposing conditions underlying NBTE. Finally, neurological complications may require the involvement of a neurologist, neurosurgeon, or interventional radiologist too.

## 10. Conclusions

Despite the rarity of NBTE, the clinical management of patients can be even more complex compared to IE. In this study, special emphasis has been placed on the diagnostic accuracy of 3D TEE, the pivotal role of multimodality imaging, and the significance of tailored therapy, particularly concerning antithrombotic approach. Finally, the establishment of a dedicated multispecialty team could provide significant advantages in managing this persistently challenging condition.

## Figures and Tables

**Table 1:** Differential diagnosis between NBTE and IE. NVE = native valve endocarditis. PVE = prosthetic valve endocarditis.

	NBTE	INFECTIVE ENDOCARDITIS
**Predisposing conditions:**	Cancer Autoimmune disorders Hypercoagulable states	Previous IE Valvular heart disease Prosthetic heart valve Congenital heart disease (CHD) Transvenous cardiac implantable electronic device Central venous or arterial catheter People who inject drugs (PWID)
**Clinical presentations:**		
Fever	−	+
Valve dysfunction	+	++
Heart failure	+	+++
Embolic events	+++	+
**Microbiology:**	Negative serial blood cultures/serology/PCR	Blood cultures/serology + in up to 70% of cases
**Echocardiography:**	Valvular thickening Verrucous, broad based, irregular vegetations, especially at valvular leaflets tip Possible false negative results if already embolized + Left-sided heart valves	Mass of variable echo density, mobility and location +++ Paravalvular complications Right-sided predilection in PWID
**CT scan:**	Cardiac CT False negatives if small vegetations Pre-operative assessment Whole-body CT Systemic embolism Neoplastic lesions detection	Cardiac CT Valvular and paravalvular lesions both in NVE and PVE (if echocardiography inconclusive) Pre-operative assessment Whole-body CT Distant lesions Sources of bacteraemia
**Brain MRI:**	Usually, small disseminated infarcts	Typically, a large infarct confined to a single vascular territory
**PET/CT scan:**	[18F] FDG uptake – for cardiac lesion	[18F] FDG uptake + for cardiac lesion
**Cardiac surgery:**	+ +++ Valve sparing	++ ++ Valve replacement
